# Feasibility study protocol of a pragmatic, randomised controlled pilot trial: membrane sweeping to prevent post-term pregnancy—the MILO Study

**DOI:** 10.1186/s13063-021-05043-9

**Published:** 2021-02-02

**Authors:** Elaine M. Finucane, Linda Biesty, Deirdre Murphy, Amanda Cotter, Eleanor Molloy, Martin O’Donnell, Shaun Treweek, Paddy Gillespie, Marian Campbell, John J. Morrison, Alberto Alvarez-Iglesias, Gill Gyte, Declan Devane

**Affiliations:** 1 University Maternity Hospital Limerick, Limerick, Ireland; 2 National University of Ireland Galway, Galway, Ireland; 3 QUESTS & School of Nursing and Midwifery, National University of Ireland Galway, Galway, Ireland; 4 Trinity College, Dublin, Ireland; 5 Coombe Women and Infants University Hospital, Dublin, Ireland; 6 University of Limerick, Limerick, Ireland; 7 HRB Clinical Research Facility Galway, Galway, Ireland; 8 Trial Forge and the Health Services Research Unit, University of Aberdeen, Aberdeen, Scotland, UK; 9 Health Services Research Unit (HSRU), University of Aberdeen, Aberdeen, Scotland UK; 10 Clinical Science Institute, National University of Ireland Galway, Galway, Ireland; 11 Galway University Hospital, Galway, Ireland; 12 Cochrane Pregnancy and Childbirth Group, Department of Women’s and Children’s Health, University of Liverpool, Liverpool, UK; 13 HRB-Trials Methodology Research Network & School of Nursing and Midwifery, National University of Ireland Galway, Galway, Ireland

**Keywords:** Feasibility, Pilot trial, SWAT, Induction of labour, Membrane sweep, Post-term

## Abstract

**Background:**

Post-term pregnancy is associated with an increased risk of maternal complications, respiratory distress and trauma to the neonate. Amniotic membrane sweeping has been recommended as a simple procedure to promote the spontaneous onset of labour. However, despite its widespread use, there is an absence of evidence on (a) its effectiveness and (b) its optimal timing and frequency. The primary aim of the MILO Study is to inform the optimal design of a future definitive randomised trial to evaluate the effectiveness (including optimal timing and frequency) of membrane sweeping to prevent post-term pregnancy. We will also assess the acceptability and feasibility of the proposed trial interventions to clinicians and women (through focus group interviews).

**Methods/design:**

Multicentre, pragmatic, parallel-group, pilot randomised controlled trial with an embedded factorial design. Pregnant women with a live, singleton foetus ≥ 38 weeks gestation; cephalic presentation; longitudinal lie; intact membranes; English speaking and ≥ 18 years of age will be randomised in a 2:1 ratio to membrane sweep versus no membrane sweep. Women allocated randomly to a sweep will then be randomised further (factorial component) to early (from 39 weeks) versus late (from 40 weeks) sweep commencement and a single versus weekly sweep. The proposed feasibility study consists of four work packages, i.e. (1) a multicentre, pilot randomised trial; (2) a health economic analysis; (3) a qualitative study; and (4) a study within the host trial (a SWAT). Outcomes to be collected include recruitment and retention rates, compliance with protocol, randomisation and allocation processes, attrition rates and cost-effectiveness. Focus groups will be held with women and clinicians to explore the acceptability and feasibility of the proposed intervention, study procedures and perceived barriers and enablers to recruitment.

**Discussion:**

The primary aim of the MILO Study is to inform the optimal design of a future definitive randomised trial to evaluate the effectiveness (including optimal timing and frequency) of membrane sweeping to prevent post-term pregnancy. Results will inform whether and how the design of the definitive trial as originally envisaged should be delivered or adapted.

**Trial registration:**

ClinicalTrials.gov NCT04307199. Registered on 12 March 2020

**Supplementary Information:**

The online version contains supplementary material available at 10.1186/s13063-021-05043-9.

## Background

Labour and childbirth are physiological processes, and for the majority of women, the onset of labour is spontaneous. However, some women will have an induction of labour. Induction of labour is the process of artificially stimulating uterine contractions to initiate the onset of labour. Approximately one in four pregnancies in the developed world will end with an induction of labour [1, 2].

Current World Health Organization guidelines note that induction of labour, as with any intervention, carries risks and advise that induction of labour is not recommended for women with uncomplicated pregnancies less than 41 weeks gestation [2]. Conversely, in response to the findings of recent studies which report that elective pharmacological induction of labour results in a lower risk of caesarean section than expectant management, the American College of Obstetricians and Gynecologists has amended their guidance to support elective induction of labour of low-risk women, having their first baby, at 39 weeks gestation [3–5]. Medical indications for induction of labour include preterm premature rupture of membranes (PPROM), intrauterine growth restriction, hypertensive disorders of pregnancy, intrauterine foetal death and post-term pregnancies [6]. Of these, post-term pregnancy is the most common [7, 8].

A pregnancy is considered to have reached full term at 37 completed week’s gestation; however, some pregnancies will continue past 41 weeks’ completed gestation and are then considered ‘post-term’ [9]. The rates of post-term pregnancy, defined as a pregnancy that has reached 42 weeks gestation from the last menstrual period (LMP), vary worldwide (0.2% in Belgium, 5.8% in the USA, 7% in Sweden) [9–12]. Birth post 42 weeks’ gestation carries an increased risk for the neonate including increased risk of meconium aspiration, neonatal acidaemia, low Apgar scores, macrosomia and neonatal death [13, 14]. The incidence of maternal complications such as severe perineal injury (third- and fourth-degree perineal lacerations) related to macrosomia, post-partum haemorrhage, chorioamnionitis and endomyometritis is increased post-term [15].

Labour may be induced using pharmacological, surgical and mechanical methods.

1. Pharmacological methods include the use of prostaglandins, such as dinoprostone administered either vaginally or intracervically; misoprostol administered orally, vaginally or intracervical; and oxytocin administered intravenously [16]. Pharmacological methods of induction of labour are not suitable for all women [17]. Reduced levels of prostaglandins are indicated in women with high parity, and the use of prostaglandins is contraindicated in cases of women with a previous caesarean section [17]. Pharmacological induction of labour increases the risk of uterine hyperstimulation [17].
2. Surgically, labour may be induced using procedures including the deliberate rupturing of the amniotic membranes known as amniotomy [18]. Amniotomy carries the risk of umbilical cord prolapse and is contraindicated when the presenting part of the foetus is not engaged in the pelvis and in women with a history of placenta praevia and vasa praevia. It also increases the risk of infection for the mother and foetus and is contraindicated in HIV-positive women [19, 20].
3. Mechanical methods of induction of labour are used to ripen and dilate the cervix encouraging the spontaneous onset of labour through manual manipulation of the cervix [21]. Mechanical methods include the use of an intracervical Foley catheter and amniotic membrane sweeping, also referred to as ‘stripping’ or ‘stretch and sweep’ of the membranes.

This study seeks to evaluate the role of membrane sweeping.

### Description of the intervention

An amniotic membrane sweep is performed with consent during a vaginal examination. It involves the clinician inserting one or two fingers into the woman’s cervix and detaching the inferior pole of the membranes from the lower uterine segment in a circular motion [22]. Membrane sweeping is a simple procedure and may be used independently or in combination with other means of induction and can be repeated multiple times.

### How the intervention might work

Amniotic membrane sweeping is used to promote the onset of labour by releasing localised prostaglandins F2α, phospholipase A2 and cytokines from the intrauterine tissues [23]. These hormones act on the cervix to augment cervical ripening potentially instigating uterine contractions. The manual stretching of the cervix may help to initiate the Ferguson reflex by releasing oxytocin thereby increasing uterine activity [23]. The aim of amniotic membrane sweeping is to soften and ripen the cervix, increasing cervical favourability and stimulate spontaneous uterine contractions potentially leading to the onset of labour and avoidance of a formal induction of labour.

### Why is this research needed?

Post-term pregnancy is by far the most common reason for induction of labour, and membrane sweeping offers a potentially low-risk method to reduce this. Membrane sweeping is a technically simple intervention and may be performed by clinicians in a community or clinical settings potentially providing significant reductions in cost [17, 24]. Recent studies have supported elective pharmacological induction of labour to lower the risk of caesarean section. However, these studies compared induction of labour to expectant management only, with none evaluating the potential effects of membrane sweeping on the process [4, 5]. Our Cochrane systematic review found that, when compared to expectant management, membrane sweeping is potentially associated with an increased rate of spontaneous onset of labour (average RR 1.21, 95% CI 1.08 to 1.34) and a lower risk of formal induction of labour (average RR = 0.73. 95% CI 0.56–0.94) when compared with expectant management [25]. It is not associated with increased rates of infection or premature rupture of the membranes and has the advantage that it may be used independently or in combination with other means of induction and can be repeated multiple times.

Guidelines by bodies including NICE [17], the Society of Obstetricians and Gynaecologists of Canada [26] and the Department of Health, South Australia [27], state that women should be offered the option of membrane sweeping at or near term. However, the optimum gestation to perform a membrane sweep to promote cervical ripening is unknown. Further, there has been a little direct comparison of the effect of multiple membrane sweeps versus a single membrane sweep to promote spontaneous labour. Internationally, guidelines have identified the need for research to clarify these uncertainties [17, 28]. In addition, our recent Cochrane systematic review found a lack of data on the optimal timing and frequency of membrane sweeping and recommended future research in this space [25]. A cost-effectiveness analysis, including an antenatal, intrapartum, postnatal and neonatal cost analysis, comparing membrane sweeping with expectant management and other methods of labour induction has not been carried out. In a time where health care providers are weighing cost-effectiveness with quality of care, this would provide invaluable data to inform health policy and is an important gap identified in our Cochrane Systematic review [25].

Clinician’s views and acceptability of membrane sweeping have been significantly under-represented in research. In addition, few studies explored women’s views of membrane sweeping. Further research to explore women’s and clinician’s experiences and views of membrane sweeping as a method of induction of labour is needed to support the clinical application of this intervention and to inform future definitive evaluations.

## Methods/design

### Trial aim and objective

The primary objective of the MILO Study is to assess the feasibility of conducting a definitive randomised controlled trial to examine the effectiveness, and optimal intensity (timing and frequency), of membrane sweeping to prevent post-term pregnancy. The study consists of four work packages.

- *WP1*: *a pilot randomised trial* assessing the feasibility of conducting a definitive trial to evaluate how often and the best time to perform a membrane sweep
- *WP2*: *health economic analysis* assessing the feasibility of conducting a trial-based economic evaluation to examine the cost-effectiveness of membrane sweeping
- *WP3*: *a qualitative study* exploring the acceptability of the trial for women and clinicians
- *WP4*: *a study within a trial (SWAT)* assessing if the point at which women are invited to take part in the trial (i.e. when should women be asked?) affects the number of women recruited to and retained in the trial

## Methods

The proposed feasibility study consists of four work packages:

### Work package 1: Pilot randomised trial

#### Methods/design

**Fig. 1:**
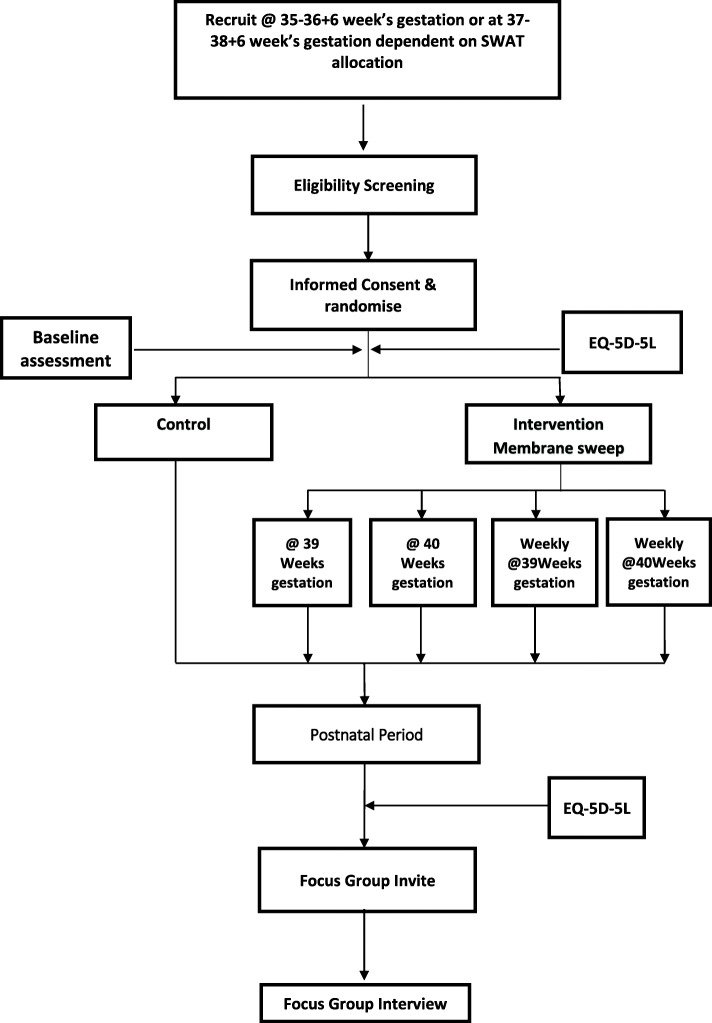
Study design

We will use a multicentre, pragmatic, parallel-group pilot randomised controlled trial with an embedded 2 × 2 factorial design (Fig. 1). This allows an examination of the feasibility of a staged ‘gated’ approach to trial analysis in a future definitive trial. For example, it allows us to evaluate the feasibility of a future trial to answer the primary question ‘is membrane sweeping effective in preventing post-term pregnancy?’ and also address the effectiveness of different timings and frequency of membrane sweeping. The advantage of using a factorial design in the MILO Study is that we can assess two individual questions simultaneously in the same population.

By utilising resources dynamically, we ensure a more efficient use of resources including sample size and time [29]. A factorial design requires a smaller sample size when compared to running two separate parallel trials resulting in reduced running and management costs and shorter time frame. The protocol has been prepared in line with the Standard Protocol Items: Recommendations for Interventional Trials (SPIRIT) guidelines (Additional file 1) [30].

#### Setting

The MILO Study will be set in the antenatal outpatient departments in two Irish maternity hospitals.

#### Participants

##### Inclusion and exclusion criteria

Pregnant women carrying a live singleton foetus ≥ 38 weeks completed gestation (gestational age will be calculated from the first day of the last menstrual period and an ultrasound examination carried out in the 2nd trimester) will be eligible. The lie must be longitudinal, presentation cephalic and amniotic membranes intact. Women must be ≥ 18 years of age on enrolment. Women will need to be able to communicate in English and give written informed consent. Women with any contraindications to a vaginal examination or vaginal birth (i.e. placenta praevia, vasa praevia, antepartum haemorrhage or undiagnosed vaginal bleeding, malpresentation, i.e. transverse lie, Herpes simplex virus with active genital lesions or prodromal symptoms) will be excluded from the MILO Study.

#### Recruitment

Written trial information will be offered to women potentially eligible for participation at 35–36 + 6 week’s gestation or at 37–38 + 6 week’s gestation, depending on SWAT randomisation (see below), during routine antenatal appointments in each site. Clinicians and/or research midwife at participating antenatal clinics will identify women who are potentially eligible to participate in the study. Women will be given an information pack that will include a letter introducing the trial and a participant information leaflet, which will inform potential participants of the background and purpose of the study, risks and benefits of participation, what participants are being asked to do, their right to withdraw and offer to answer any questions they have relating to the study. This will be followed up at the 39-week antenatal visit when the researcher will invite eligible women to participate.

#### Obtaining informed consent

At the 39-week antenatal visit, the potential for inclusion to the trial will be checked by the attending midwife and/or research midwife. The trial will be explained, and questions potential participants might have will be answered. Eligible women will be asked to participate at this time, and written informed consent will be obtained from women agreeing to participate.

#### Randomisation and allocation concealment

Randomisation to intervention and control will be at the level of the individual, i.e. individual randomisation, stratified by parity and centre. Randomisation is on 2:1 ratio; that is, for every two women randomised to the intervention arm (sweeping intervention), one will be randomised to the control arm (usual care). Women in the intervention group will further be randomised in a ratio of 1:1 to the factorial design. The random allocation sequence will be generated using a computer-generated random number list. Random permuted blocks of sizes 6 and 12 will be used to determine the group allocation. Randomisation will be stratified by (a) parity to ensure appropriate representation of primiparous and multiparous women to each group and (b) centre using a separate block randomisation list for each of the two centres. Block sizes will be concealed until completion of the trial.

To ensure concealment of allocation, randomisation will be done electronically using web-based random allocation based on random sequence generation detailed above. The enrolling midwife will log stratification factors with the randomisation service through a web interface after which he/she will be informed of the allocation (usual care or group allocation in the 2 × 2 factorial design) and the unique study ID number, which will be documented on the consent form.

#### Blinding

Clinicians performing a membrane sweep cannot be blinded, and it is not feasible to genuinely blind membrane sweeping for women. Therefore, neither clinicians administering the intervention nor women will be blinded to the group assignment. Data will be reviewed by two assessors blinded to the group allocation.

#### Intervention

Amniotic membrane sweeping is defined as the manual detachment of the inferior pole of the amniotic membranes from the lower uterine segment [22]. This is performed with consent by a clinician digitally through a circular motion during a vaginal examination. If the cervical os is closed, massage of the cervix will be accepted.

Women will initially be randomised in a 2:1 ratio to:

- Membrane sweep (2) versus no membrane sweep (1)

Those allocated to the intervention group will then be further randomised in a factorial fashion to A, B, C or D (Table 1):

A. Membrane sweep at 39 weeks’ gestation only
B. Membrane sweep at 40 weeks’ gestation only
C. Membrane sweep at 39, 40 and 41 weeks’ gestation or until the onset of labour
D. Membrane sweep at 40 and 41 weeks’ gestation or until the onset of labour

**Table 1:** Allocation of the intervention group

	Sweeping at 39 weeks	Sweeping at 40 weeks
Single membrane sweeping	A	B
Weekly membrane sweeping (up to 41 weeks or until onset of labour)	C	D

#### Control group

Women in the control arm will not receive a membrane sweep and will receive usual care (as defined by local hospital protocols and vaginal examination to determine Bishop score only). Usual care in both sites is the same and includes women attending for routine antenatal clinic appointments monthly up to week 32, fortnightly to week 38 and weekly to week 42. Women will be offered induction of labour at approximately 41 weeks’ gestation and labour induced in most women prior to 42 weeks’ gestation. We will identify any intricacies of usual care that might be present in each site but that might not become apparent outside of a research context as part of the study through the mapping usual care pathways. Other than randomisation to an intervention group or a control group, all women will receive usual care as defined by local hospital protocols. Participating in this trial will not alter the intrapartum or postnatal care pathway for the woman or her infant.

#### Withdrawal from trial/treatment or protocol deviation post-randomisation

If a woman decides to leave the trial after randomisation, she will be withdrawn from the trial and will receive usual maternity care as defined by local hospital policy. The same strategy will be implemented for protocol violations. Randomisation will take place immediately prior to the commencement of the intervention to try and mitigate these events. The pilot trial will use intention-to-treat (ITT) data analysis. If a woman withdraws from the trial, we will try to obtain consent to collect data relevant to the study and/or routine follow-up data. Information and communications will be recorded in the trial database.

#### Clinician training

All necessary midwives and obstetricians will receive the MILO training programme, which will include training on how to perform a membrane sweep per trial definition and training on the study protocol to enable them to support recruitment of women to the study, answer any questions women or their partners may have, support the taking of informed consent and randomisation of women. Recruitment will be supported by on-site research midwife, and training of clinicians will be dependent on the tasks they undertake. To enhance validity, reliability and generalisability of the intervention, special consideration will be given to the training of clinicians performing a membrane sweep to ensure treatment fidelity. We will develop a standardised intervention manual, and prior to the intervention start date, all clinicians who might perform a sweep will receive the manual. In addition, all relevant clinicians will receive training in the form of a tutorial video and hands-on training from an experienced trainer. This training session will teach a standardised protocol for the intervention. Adherence to this protocol will be monitored throughout the trial by the research midwife and the trial project manager.

#### Outcome measures

We will collect the following outcome data:

#### Primary outcomes

The following are the outcomes relating to feasibility assessment:

1. Recruitment: evaluation of the number and percentage of eligible women who are recruited and randomised to the study. Assessed by study-specific checklists.
2. Retention: evaluation of the number and percentage of eligible women who are randomised, take part in and adhere to the study protocols. Data will be extracted from routinely collected data.
3. Adherence with the trial interventions: evaluation of adherence with the trial interventions and reasons for non-compliance assessed by study-specific checklists. Data will be extracted from routinely collected data and focus group interviews with clinicians and participants at 6 weeks post-intervention.
4. Evaluation of the randomisation process: evaluation of effective allocation of participants to the intervention/control group assessed by study-specific checklists and evaluation of the randomisation protocol throughout the randomisation period.
5. Evaluation of attrition rates: evaluation of attrition rates assessed by study-specific checklists. Data will be extracted from routinely collected data.
6. Evaluation of the types of attrition: evaluation of the types of attrition assessed by case report forms. Data will be extracted from routinely collected data.
7. Evaluation of the data collection process through study-specific checklists: evaluated, statistically and narratively, by assessing the completeness of the outcome measurements at baseline and postnatal (6 weeks) through study-specific checklists. Researchers will manually examine the data collected. They will assess the proportion of complete data collection forms, the quality of data collected and the applicability of this data in facilitating pilot trial outcomes.
8. Estimate the main effect of individual intervention components and their interactions: estimates (with measures of uncertainty) of the main effect of individual intervention components and any interaction effect between the main effects of the embedded factorial design will be assessed and reported using regression analysis.
9. Evaluation of the data analysis process: as this is a feasibility study, formal hypothesis testing will not be undertaken. Researchers will manually examine the data collected. Evaluation of the data analysis process will be undertaken through the assessment of gaps and limitations to the analysis process measured by study-specific checklists. Findings will be reported through descriptive statistics and graphical summaries.
10. Evaluation of the EQ 5D: assessment of the mechanism of, timing of and delivery of the EQ 5D through study-specific checklists.
11. Feasibility of cost analysis process through analysis of study-specific documentation: assessment of data collection tools to undertake cost-effectiveness analysis through study-specific documentation. Researchers will manually examine the data to assess the mechanism of, timing of and delivery of the cost analysis tools.
12. Feasibility of the cost-effectiveness analyses: assessment of the mechanism and utilisation of the incremental cost-effectiveness ratio (ICER), through study-specific checklists.

#### Clinical outcomes

This study will also collect clinical and adverse outcome data that are likely to be collected in the future definitive trial. This is done not to evaluate the clinical effectiveness of membrane sweeping within a pilot trial but to test the outcome collection processes and to help inform the sample size estimates for and safety of a future definitive study. Data will be extracted from routinely collected data. These outcomes are as follows.

##### Primary outcome (of future definitive trial)

The primary outcome is the number of participants achieving a spontaneous onset of labour.

##### Maternal secondary outcomes

- Number of participants who underwent an induction of labour: formal induction of labour using pharmacological or surgical methods.
- Number of participants achieving a spontaneous vaginal birth: spontaneous vaginal birth.
- Instrumental birth: vaginal birth which is assisted with the use of instruments.
- Caesarean section: birth which is achieved through the surgical procedure caesarean section.
- Post-partum haemorrhage ≥ 500 ml: blood loss ≥ 500mls within the first 24 h of the birth of a baby.
- Ante-partum haemorrhage requiring hospital admission: bleeding from the genital tract, from 24 + 0 weeks of pregnancy and before the birth of the baby.
- Uterine hyperstimulation with/without foetal heart rate (FHR) changes: uterine hyperstimulation defined as uterine tachysystole (more than five contractions per 10 min for at least 20 min) and uterine hypersystole/hypertonicity (a contraction lasting at least 2 min). These may or not be associated with changes in the foetal heart rate pattern (persistent decelerations, tachycardia or decreased short term variability) [31].
- Serious maternal death or morbidity (e.g. uterine rupture, admission to intensive care unit, septicaemia).
- Epidural analgesia: introduction of a local anaesthetic into the epidural space of the vertebral canal.
- Augmentation of established labour: the stimulation of uterine contractions using pharmacologic methods or artificial rupture of the membranes to increase their frequency and/or strength following the onset of spontaneous labour or contractions following spontaneous rupture of membranes.
- Pyrexia in labour: pyrexia that developed any time after the onset of labour.
- Uterine rupture: all clinically significant ruptures of unscarred or scarred uteri. Trivial scar dehiscence noted incidentally at the time of surgery will be excluded [31].
- EQ 5D-5L: EuroQol EQ 5D-5L survey instrument.

##### Neonatal secondary outcomes

- Serious neonatal morbidity (e.g. seizures, birth asphyxia defined by trialists, neonatal encephalopathy, disability in childhood, proven and suspected neonatal sepsis)
- Apgar score < 7 at 5 min
- Cord PH < 7.20: umbilical cord blood gas test
- Neonatal encephalopathy: (severity of hypoxic-ischaemic encephalopathy assessed using Sarnat staging: (i) stage 1 (mild)—hyper-alertness, hyper-reflexia, dilated pupils, tachycardia and absence of seizures; (ii) stage 2 (moderate)—lethargy, hyper-reflexia, miosis, bradycardia, seizures, hypotonia with weak suck and Moro reflexes; and (iii) stage 3 (severe)—stupor, flaccidity, small to mid-position pupils which react poorly to light, decreased stretch reflexes, hypothermia and absent Moro reflex)
- Perinatal death: (the perinatal period is defined as ‘commences at 22 completed weeks (154 days) of gestation and ends seven completed days after birth.’ [32])
- Admission to neonatal intensive care unit (NICU) or equivalent

##### Maternal and neonatal process outcomes

- Length of time from membrane sweep to the birth of a baby
- Length of time from formal induction of labour to the birth of a baby
- Overall length of maternal hospital stay
- Length of infant stay in NICU or equivalent

Baseline data to include age, obstetric history, parity and Bishop Score will be collected for all participants on the first vaginal exam at the time of randomisation.

#### Statistical methods and analysis

##### Sample size for pilot trial

As this is a pilot trial and not designed to evaluate the clinical effectiveness, we will not undertake a formal power analysis for sample size. We will seek to recruit 66 women per clinical site (132 women in total) over a 6-month period beginning in July 2020. This target represents 10% of that required for the definitive trial (see below) and is greater than that recommended as the minimum sample sizes for pilot studies [33]. Data obtained from this study will inform the power analysis for a definitive trial.

##### Sample size for definitive trial

The primary outcome for the definite trial will be the spontaneous onset of labour. National data demonstrate a spontaneous onset of labour rate of 54% in women without routine membrane sweeping to prevent post-term pregnancy. A sample size of 910 in the intervention arm and 455 in the control group (2:1 randomisation, 1365 total) will have sufficient power (at > 80%) to detect a 15% relative increase in the primary outcome measure, that is from 54% without membrane sweeping to 62% with membrane sweeping. These calculations assume alpha of 0.05 and the test is 2-tailed.

#### Criteria for progressing to the main definitive trial

The criteria for progressing to a future definitive trial are based on the primary feasibility objectives of the pilot trial. The pilot will be deemed suitable to continue to definitive trial when the following are achieved:

- Recruitment
  At least 30% of eligible women agree to participate in the trial and 130 women are randomised.
- Completeness of outcome data
  Complete clinical outcome data that would be collected in the main trial collected from at least 90% of pilot trial participants.
- Clinician willingness to participate
  At least 70% of participating clinicians within the two pilot sites agree that they would be happy to implement the MILO Study. Clinician’s views, experiences and acceptability of the MILO Study will be explored within focus group interviews.

Given the primary objective of the MILO Study is to assess the feasibility of conducting a definitive randomised controlled trial, we will evaluate recruitment and retention, adherence to the MILO protocol and reasons for non-compliance, and clinicians and women’s views, experiences and acceptability of the MILO Study. In the event the MILO Study does not meet the above criteria, these results will inform whether and how the design of the definitive trial as originally envisaged should be delivered or adapted.

#### End of trial discontinuation criteria

##### Individual participant

- Withdrawal of informed consent
- Development of exclusion criteria or other safety reasons during the study
- Incorrect enrolment or randomisation of the participant (data retained for purpose of analysis)
- Unanticipated adverse event (consideration given to whether the participant should be discontinued)

##### Recruitment centre

- Not reaching pre-specified recruitment targets (at least 30% of eligible women agree to participate in the trial and 130 women are randomised)
- Systemic non-adherence to protocol

##### Trial

If IDMC requires termination of the study, e.g. futility analyses show no benefit to ongoing recruitment.

For the woman, the pilot trial is considered ended on discharge from the maternity hospital. For the infant, the pilot trial is considered ended on discharge from the maternity hospital or from the neonatal unit.

#### Co-enrolment

Women enrolled in this trial may not take part in other interventional trials during the antenatal or intrapartum period evaluating induction of labour or cervical ripening.

#### Data collection, management and analysis

A data management plan will be completed outlining the data management process prior to the collection and analysis of study data.

##### Data collection forms

Paper forms will be used in each participating site to confirm eligibility prior to randomisation and to record informed consent. Data will be collected from the participating maternity hospitals using paper-based case report forms (CRFs). Data will be collected retrospectively by the research midwife in each site. The participating sites will collect the woman’s hospital number, and this may be used in the process of collecting missing data. With the exception of the on-site research midwife, the research team will only have access to a unique identifier for the participant for the purpose of data management. Clinical outcomes are recorded in a woman’s health care records, i.e. gestation, number of sweeps performed and gestation of woman at the time of membrane sweep, hyperstimulation, mode of delivery, analgesia, Apgar scores, length of stay and infant admission to NICU. This retrospective data from the clinical notes and the CRF are considered source data.

##### Storage of data

All identifiable information will be held on a secure, password-protected database accessible only to pre-defined personnel. Paper forms with identifiable information will be held in secure, locked filing cabinets. Personal data collected during the trial will be handled and stored in compliance with the 2018 General Data Protection Regulation (GDPR). Participants will be identified by a given code only. Data from the randomisation paper form, CRF and outcome data collected from women’s notes will be entered onto a purposefully designed Excel database, within 7 days of the woman’s discharge, by the research midwives. All entries to the database will be recorded and dated and each version archived to ensure good clinical practice. Entered data will later be double-checked against original forms for accuracy. All paper forms and data checking records will be securely archived after completion of trial as per requirements under the General Data Protection Regulation EU 2016/679. Direct access to the source data/documents will be required for trial-related monitoring by authorised personnel only.

##### Data analysis

All data will be analysed and reported in accordance with the 2010 CONSORT Extension Statement for the reporting of Pilot and Feasibility studies [34]. As this is a feasibility study with a relatively small sample size, formal hypothesis testing is not appropriate; rather, the purpose of any analyses will be to generate estimates to inform the planning of the definitive future trial. Suitable descriptive statistics and graphical summaries will be used to summarise participant characteristics. Means and standard deviations will be used for continuous variables and counts and percentages for categorical variables. Estimates of variation in main effects will be used to inform future sample size calculations. Estimates (with measures of uncertainty) of any interaction effect between the main effects of the embedded factorial design will also be undertaken. These will refine the design characteristics of the future definitive trial.

#### Reporting serious adverse events

Membrane sweeping has been found to be a low-risk intervention with no increased risk of infection or premature rupture of membranes. All adverse events will be reported to the trial team and recorded on the woman’s CRF. In addition, adverse events will be documented in the participant’s health records. An expected adverse event is discomfort during the membrane sweeping procedure.

### Work package 2: Health economic analysis

As this is a pilot trial, we will not undertake a formal economic evaluation. The health economic analysis will assess the feasibility of conducting a trial-based economic evaluation to examine the cost-effectiveness of membrane sweeping relative to expectant management and other methods of induction of labour to prevent post-term pregnancy. The basic tasks of economic evaluation are to identify, measure, value and compare the costs and outcomes of the alternative strategies being considered. The pilot study explores the feasibility of conducting an economic evaluation in this context and will seek to inform the design of the economic evaluation to be conducted alongside the definitive RCT. Evidence collected on resource use and outcome measures alongside the pilot RCT will provide the basis for the analysis. With respect to costing, a healthcare service perspective will be adopted, and the study will seek to identify the healthcare resource items that are relevant in this case. In particular, resource use associated with the implementation of the membrane sweeping intervention and the alternative expectant management and pharmacologic control strategies will be identified, measured and costed. In addition, other resource use over the course of the pregnancy in respect of antenatal, intrapartum and postnatal care will be identified, measured and costed. Unit costs will be identified and applied to convert data on resource use to resource costs, and total cost variables will be calculated. The pilot will involve the development and testing of appropriate data collection tools to undertake this process. For the pilot cost-effectiveness analysis, the alternative strategies will be compared on the basis of the clinical outcome data identified in the pilot RCT. This will inform costing models for the future definitive trial. For the cost-utility analysis, quality-adjusted life years (QALYs) will be modelled using the EuroQol EQ 5D-5L survey instrument. The pilot study will explore the feasibility, suitability and appropriate timing and delivery of the EQ 5D-5L in this context. To complete the pilot study, an incremental analysis will be conducted to model the mean costs and mean effect comparisons of the membrane sweeping intervention relative to the control strategies, which will inform the analysis models in the definitive trial. Univariate and multivariate sensitivity analyses, in addition to probabilistic methods through the estimation of cost-effectiveness acceptability curves, will be employed to explore uncertainty.

### Work package 3: Qualitative descriptive study

O’Cathain et al. [35] note the contribution qualitative research can make to feasibility studies by exploring uncertainties associated, for example, with interventions, trial methodology and outcome measures, prior to the conduct of a definite trial. Drawing on the guidance O’Cathain et al. [35] offer for such qualitative work, this feasibility study will include a qualitative descriptive study to explore the acceptability and feasibility of the MILO Study. This will include the clinician and women’s views of membrane sweeping, relevance and acceptance of the clinician training programme, and potential barriers and enablers to recruitment for a definitive trial.

#### Design

This work package will use a qualitative descriptive study design. Qualitative descriptive studies aim to explore and to understand the perspectives of those directly involved in certain processes or phenomenon [36], and so this design lends itself well to an exploration of the views of key stakeholders participating in the MILO Study.

#### Participants

Purposeful sampling will be used. Up to 10 women per clinical site (this target represents 15% of MILO participants) and all clinicians participating in the pilot trial will be invited to participate in the focus group interviews. All potential participants will be contacted via letter when the last trial participant has been discharged from the maternity unit and invited to participate in one of two focus groups based on their geographical location. All letters will make clear the number of participants required. The experiences and views of women across the control and intervention groups will be explored in order to provide an insight into all aspects of the feasibility study.

#### Data collection

Data will be collected via focus group interviews carried out in each participating site with two focus groups for each of clinicians and women stakeholders (four focus groups in total). The sessions will be led by an experienced qualitative researcher. A topic guide, informed by the purpose of the study and by the literature, will be used to guide the focus groups.

#### Data analysis

Focus groups will be audio-recorded, and recordings will be transcribed verbatim and entered into Nvivo. A pseudonym will be given for each participant and will be used on all transcripts of interviews. Data will be analysed using the framework method, a method of analysis for qualitative data described by [37]. Identified themes will inform the design of a future definitive trial.

### Work package 4: Study within a trial

#### Background

Adequate recruitment of trial participants is essential to the success of all trials. Yet, two thirds of trials will not complete recruitment within their stated time frame [38]. Pregnant women in particular remain underrepresented in clinical research, and the recruitment of pregnant women to trials has proved challenging [39]. A 2018 Cochrane systematic review examining the methods to improve recruitment to randomised controlled trials found a distinct knowledge gap in evidence-based recruitment strategies [40]. A study within a trial (SWAT) provides an opportunity to increase the evidence base about trial processes (e.g. recruitment and retention).

#### Aim

To evaluate the effect of the timing of the invitation to women to take part in the trial on recruitment and retention.

#### Design

Cluster randomised trial.

#### Setting

As per host pilot trial

#### Participants

As per host pilot trial

#### Intervention

Group 1: Participant recruitment at 35–36 weeks + 6 days gestation

Group 2: Participant recruitment at 37–38 weeks + 6 days gestation

#### Randomisation

To minimise the impact of the embedded SWAT on the design and conduct of the definitive trial, randomisation to the different timings of recruitment will be conducted at the site level, i.e. site randomisation. Each of the 2 sites will be randomised to recruit women from group 1 or group 2.

#### Recruitment

##### Identifying potential participants

Clinicians at participating antenatal clinics will identify potential participants that meet the study inclusion criteria. Written trial information will be offered to women potentially eligible for participation at 35–36^+6^ week’s gestation OR 37–38^+6^ week’s gestation, dependent on-site randomisation in the SWAT, during routine antenatal appointments. Women will be given an information pack, which will include a letter introducing the trial and a participant information leaflet, which will inform participants of the background and purpose of the study, risks and benefits of participation, what participants are being asked to do, their right to withdraw and offer to answer any questions they have relating to the study. This will be followed up at the 39-week antenatal visit when the researcher will invite eligible women to participate.

#### Obtaining informed consent

At the 39-week antenatal visit, potential for inclusion to the trial will be checked by the attending clinician and/or research staff. The attending clinician and/or research staff (we expect this will be the researcher unless at the request of clinical staff) will be available to explain the trial and answer any questions potential participants might have. Eligible women will be asked to participate at this time and written informed consent will be obtained from women agreeing to participate.

#### Outcomes

##### Primary outcomes

- Evaluation of randomisation, allocation and concealment processes through focus group interviews and data extracted from routinely collected data
- Estimate variable parameters to inform sample size for definitive trial, including standard deviation of the outcome measure

##### Secondary outcomes

- Proportion of eligible women recruited. Data will be extracted from routinely collected data.
- Proportion of recruited women that complete trial. Data will be extracted from routinely collected data.

#### Sample size

##### As per host trial

Table 2 outlines the schedule of enrolment, interventions and assessments within The MILO Study.

**Table 2:** Schedule of enrolment, interventions and assessments within the MILO Study

Time point	Study period					
	35–36^**+6**^ OR 37–38^**+6**^ weeks gestation (*dependent on SWAT allocation*)	39 weeks gestation	40 weeks gestation	41 weeks gestation	Postnatal period (*after last study participant is discharged from maternity*)	Postnatal period (*6 weeks after the last participant has given birth*)
**Eligibility screen and written information**	X					
**Informed consent**		X				
**Allocation**		X				
**Intervention**						
**Group A**		X				
**Group B**			X			
**Group C**		X	X	X		
**Group D**			X	X		
**Qualitative study written information**					X	
**Focus group interviews**						X
**Qualitative study informed consent**						X
**EQ-5D-5L evaluation**		X			X	

#### Ethical and safety considerations

##### Independent data monitoring committee

We will establish an independent data monitoring committee (IDMC) to monitor data emerging from the MILO Study. The IDMC will meet regularly (as required) to assess trial progress based on independent trial data.

#### Ethical approval

The MILO Study will be conducted in full conformance with the principles of the Declaration of Helsinki and to Good Clinical Practice (GCP) guidelines. We have sought and obtained ethical approval from both study sites (University Maternity Hospital Limerick and the Coombe Women and Infants University Maternity Hospital).

## Discussion

Conducting a feasibility study prior to a definitive trial potentially reduces the risk of research waste through evaluation of trial processes such as recruitment and retention, randomisation, intervention compliance and data management. In 2009, Chalmers and Glasziou, estimated that 85% of all health research is being avoidably wasted [41]. Poor question choice, inappropriate trial design and inaccurate reporting of results have all contributed to research waste [42]. Worldwide, significant public funding is allocated to support biomedical and clinical research [43]. In the USA, the National Institutes of Health (NIH) invests approximately US$39.2 billion a year in medical research [44]. In 2015/2016, the National Institute for Health Research (NIHR) invested £247 million [45]. Demands to improve the efficiency and effectiveness of public expenditure have increased pressure on publicly funded research budgets. For clinical trials to be sustainable, methods to reduce costs and increase productivity must be prioritised. The publication of feasibility study findings inform the design of definitive trials reducing the risk of future research waste.

Induction of labour is often viewed as a ‘common’ intervention with approximately one in four pregnancies ending in an induction of labour [2]. Membrane sweeping potentially offers a low risk, effective intervention to prevent a formal induction of labour that is routinely offered to pregnant women. However, despite this, its effectiveness (including optimal timing and frequency) to prevent a formal induction of labour is unknown [25]. With the MILO Study, we will evaluate the feasibility of conducting a definitive randomised trial to assess the safety and effectiveness of membrane sweeping in preventing a formal induction of labour in women at or near term.

To maximise sample size efficiency and cost-effectiveness, we chose to utilise a factorial design which will assess these questions simultaneously using the same population [29]. The MILO Study will be conducted in the antenatal department of two large maternity hospitals. Mindful of minimising the impact of the trial on the participating clinical sites, we designed all components of the study, including intervention timings, to align with the usual care pathway of pregnant women attending these hospitals. Although when developing the methodology for the MILO Study we did not expect to conduct the trial during a pandemic, this design has proved advantageous as it ensures that women taking part in the trial will not be required to attend additional antenatal appointments, while also maximising the potential population from which the study will recruit.

The onset of the COVID-19 pandemic caused significant changes to clinical practice, presenting unforeseen challenges to clinical trials. The MILO Study, like many trials, has had to pause recruitment, and we have adapted its design to facilitate and overcome these challenges. These adaptations are focused on minimising avoidable face-to-face contact.

Initially, during the recruitment of potential participants, we had planned to provide a private room in which to answer queries on the MILO Study. However, due to the current national guidelines, this will no longer be feasible. To support recruitment and informed consent, we will now offer women, through a letter contained in the information pack, the option to engage with the research team through scheduled calls. Women will not be asked to provide consent during these calls; the purpose of these calls is to facilitate further information on the MILO Study if requested. In addition, we had planned to offer the option of face-to-face or online focus group interviews with women and clinicians (WP3—qualitative descriptive study). Interviews will now be facilitated online or by phone, either as a one-to-one meeting or within a group setting.

In conclusion, the findings of the MILO Study, including the views of women and clinicians, will inform the design of a future definitive trial to examine the effectiveness, and optimal intensity (timing and frequency), of membrane sweeping, a common intervention in maternity care, to prevent post-term pregnancy.

## Trial status

The MILO Study will begin recruiting in February 2021.

It is anticipated that recruitment will be completed in September 2021.

Protocol version: 05 January 2021, version 1.2

## Supplementary Information

**Additional file 1.** : SPIRIT 2013 Checklist: Recommended items to address in a clinical trial protocol and related documents.

## Data Availability

The MILO Study has not yet begun recruiting. All data and materials will be available from the corresponding author on reasonable request.

## Abbreviations

AE: Adverse event
AR: Adverse reaction
IOL: Induction of labour
PPH: Post-partum haemorrhage
PROM: Preterm premature rupture of membranes
PPROM: Preterm premature rupture of membranes
SVD: Spontaneous vaginal delivery
APH: Antepartum haemorrhage
CS: Caesarean section
SWAT: Study within a trial
